# “Bird’s” Treatment of Prolapsus Uteri

**Published:** 1864-09

**Authors:** W. B. Slayter

**Affiliations:** Chicago, Graduate Royal College of Physicians of England, Royal College of Surgeons of London and Dublin. Late House-Surgeon of Westminster Hospital, London


### "BIRD'S" TREATMENT OF PROLAPSUS UTERI.

By W. B. SLAYTER, M. D., of Chicago,
*Graduate Royal College of Physicians of England, Royal College of Surgeons of London and Dublin. Late House-Surgeon of Westminster Hospital, London.*

One of the most loathsome and troublesome diseases from which a woman can suffer, is Prolapsus Uteri. It not only causes pain and bodily suffering, but makes her life one of misery, and sometimes positively unbearable. Various plans of treatment have been pursued, but in the majority of cases are of no avail. Little can be done except to give temporary relief. The mode of treatment devised by Drs. Golding and Frederick Bird, of London, for the permanent relief of the disease, has been practiced with marked success by the profession in Europe, and deserves the careful consideration of the profession in this country.

The operation consists in returning the Uterus to its natural situation, and then cauterizing the upper part of the Vagina; it is performed in the following manner: The patient first having been placed fully under the influence of chloroform, and the Uterus returned to its natnral situation; the walls of the Vagina are widely separated and protected by metal spatulas held by an assistant; a cautery iron having a cone shaped top about half an inch long, and bent at right angles with the handle, and which has been made red hot, is then carefully inserted into the vagina, and the upper part of the passage around the cervix uteri is gently cauterized, forming an eschar about half an inch wide. A pledget of lint, well oiled, is then inserted, and the patient kept quiet in bed for two or three days. The slough comes away in a short time, the ulcerated surface heals up, and the cicatrix resulting, as is well known, rapidly contracts, and so prevents the Uterus again coming down.

This treatment is, of course, only applicable to those cases where there is no chance of future child-bearing for if the patient became pregnant it would seriously endanger her life, as it would be an impossibility for a child to pass the hard, dense cicatrix in the Vagina.

Many cases have been published in the British Medical Journals, in which the operation has been successfully employed, and several cases which have been under treatment at the Westminster Hospital, and the details of which I have published in the *London Lancet*, fully show the benefits resulting from it. The advantages of the operation are too palpable to require mentioning; it is, however, reasonable to suppose that if the cautery were applied too extensively, and without due caution, serious inflammation and its consequences might arise; but in all those cases which have come under my notice, no serious symptoms of any kind have arisen, and in from ten days to a fortnight the patients have been able to go about, and resume their occupations as usual, without the slightest inconvenience. I have seen several cases a considerable time after the operation had been performed, and they have had no return whatever of the Prolaps.

The two principal points in the operation, to be attended to are, 1st. To see that the walls of the Vagina are thoroughly protected before the cautery iron is introduced, and 2d. That the eschar is not more than half an inch wide, and does not implicate anything but the mucous membrane of the Vagina. The pain after the operation, as a rule, is slight, although in some cases it is considerable, but is readily controlled by the liberal administration of opiates.

In conclusion, I would call on the profession to give this operation their earnest consideration, and in those cases where other remedies have failed, to give it a trial, as I am convinced that in many well chosen cases they will be thoroughly satisfied with it.

